# Semaglutide - properties, action and chromatographic analysis

**DOI:** 10.1007/s40200-025-01711-8

**Published:** 2025-09-08

**Authors:** Barbara Wasilewska, Anna Petruczynik

**Affiliations:** Department of Inorganic Chemistry, Medical University of Lublin, Chodźki 4a, Lublin, 20-093 Poland

## Abstract

**Objectives:**

This review assessed the selected information on semaglutide’s activity, its potential for the treatment of various diseases, and its pharmacokinetics. It is intended as a guide for future research. Chromatographic procedures used for the determination of semaglutide in various biological samples were also reviewed.

**Methods:**

A comprehensive review of the literature was conducted by searching scientific databases including PubMed, Scopus, Web of Science, and Google Scholar. The search was performed using keywords such as diabetic, type of diabetics, impact of diabetic glucagon-like peptide, DPP-4 inhibitors, GLP-1 agonists, semaglutide and weight loss, semaglutide and obesity, semaglutide and diabetic retinopathy, semalutide and mood, semaglutide and mood disorder, semaglutide and fertility, semaglutide and thyroid, semaglutide and inflammation, semaglutide and cardiovascular system, semaglutide and imapct on heart, semaglutide and neuroprotection, semaglutide and pancreatitis, safety of semaglutide, semaglutide and side effects, semaglutide and contraindication, and semaglutide analysis by liquid chromatography.

**Results:**

Semaglutide is the most potent glucose-lowering glucagon-like peptide (GLP-1) analogue and is widely used in the treatment of type 2 diabetes. Semaglutide increases the secretion of insulin from pancreatic β-cells and supresses glucagon release from pancreatic α-cells. Due to its effects on appetite regulation, it is also used to treat obesity in many countries. However, due to the slimming properties of the drug, semaglutide is often abused by non-diabetics, non-obese individuals, and young people. Recently, numerous investigations have been conducted to better understand the mechanism of action, as well as the advantages and disadvantages of using semaglutide. It is also very important to develop sensitive and accurate methods for detecting this drug in various biological samples collected from patients.

**Conclusion:**

Semaglutide is increasingly used of for the treatment of type 2 diabetes; however, its misuse for weight loss is also increasing. Further research is required to confirm the benefits of using semaglutide and to optimize treatment strategies for diverse patient populations.

## Introduction

Diabetes is one of the most common metabolic and civilizational diseases. Polydipsia, dry skin, frequent urination, fatigue, blurred vision, unexplained weight loss and even depression can be symptoms of excessively high levels of sugar in the human bloodstream [1, 2].

Dysfunctional secretion of pancreatic hormone insulin plays a main role in this disorder. A deficiency of insulin or a lack of sensitivity of peripheral receptors to insulin lead to hyperglycemia. Glucose is overproduced and underutylized as an energy source [1]. There are several types of diabetes. The most common forms include:

- type 1,
- type 2,
- gestational diabetes,
- other specific types of diabetes, such as latent autoimmune diabetes in adults (LADA), and maturity-onset diabetes of the young (MODY).

- Type 1
  Type 1 diabetes (also known as juvenile diabetes) occurs in children and teenagers. Initial symptoms appear very quickly and progress rapidly [1]. This form of diabetes has an autoimmune cause. Endogenous antibodies damage pancreatic beta cells that are responsible for producing insulin. An insufficient amount of this hormone in the bloodstream leads to hyperglycemia. Diabetics use natural or synthetic insulin as the only method of medicinal treatment.
- Type 2
  Type 2 diabetes usually appears in adults. A lack of physical activity, obesity, and a diet high in sugar content are the main contributing factors to elevated levels of sugar in the blood [1]. This type of diabetes is connected with insulin resistance. Insulin resistance occurs when human cells respond inadequately to insulin, impairing their ability to efficiently absorb and store glucose from the bloodstream. Treatment begins with lifestyle modifications, followed by antidiabetic drugs and insulin.
- Gestational diabetes
  Gestational Diabetes can develop during pregnancy. Between 30% and 70% of gestational diabetes is diagnosed in early pregnancy (early gestational Diabetes is defined by hyperglycaemia occurring before 20 weeks of gestation) [3]. Early gestational diabetes is associated with worse pregnancy outcomes compared to women diagnosed with late gestational diabetes (hyperglycaemia typically from 24 weeks to 28 weeks of gestation). In most instances, the condition resolves after childbirth.
- LADA (Latent Autoimmune Diabetes in Adults)
  Latent autoimmune Diabetes in adults exhibits characteristics of both type 1 and type 2 diabetes. It results from an autoimmune reaction (autoantibodies targeting pancreatic beta cells), but it develops much more slowly than type 1 diabetes [4]. Five serum autoantibodies are indicative of humoral immunity in LADA: glutamic acid decarboxylase autoantibody (GADA) (one of the most sensitive immunological criteria for diagnosing LADA), pancreatic islet-cell antibodies (ICA), insulinoma associated-2 autoantibodies (IA-2 A), autoantibodies against insulin (IAA), and an association of zinc transporter-8 autoantibody (ZnT8A).
- MODY (Maturity-Onset Diabetes of the Young)
  MODY starts in adolescence or early adulthood and is inheritted in an autosomal dominant way. As a result, insulin secretion by Pancreatic beta cells is impaired due to mutations in single genes. This kind of Diabetes has 14 subtypes with differences in prevalence and associated complications. The most common subtype is MODY 3, followed by MODY 2 and MODY 1. Diabetics are treated with a low or highdose of sulfonylurea derivatives and eventually insulin [5].
  Untreated diabetes is connected with numerous negative consequences. Acute diabetes complications can be Life-threatening. People with type 1 Diabetes are at a risk of ketoacidosis. This occurs when insulin levels are too low and the human body cannot use glucose for energy, forcing it to break down fat instead. This process eventually releases ketones, which can accumulate in the blood and make it acidic. The hyperosmolar hyperglycemic state mainly affects people with type 2 diabetes. Very high blood sugar levels over a prolonged period lead to severe dehydration [6]. Long-term high glucose levels primarily damage blood vessels and nerves. Damaged blood vessels increase the risk of heart attack and stroke. Other long-term diabetes complications include: nephropathy, retinopathy, neuropathy, gastroparesis, skin infections, and diabetic foot [1, 7]. Diabetic foot refers to ulcers that form on the dorsal or plantar part of the foot. Diabetic foot ulcers can worsen and lead to tissue necrosis, infection, and ultimately, amputation [8].

## Glucagon-like peptide (GLP-1)

GLP-1 is a peptide hormone composed of of 30 or 31 amino acids [9]. It is produced in two bioactive forms consisting of glycine-extended GLP-1 and amidated GLP-1 via the post-translational processing of proglucagon by proprotein convertase subtilisin-kexin type 1 (PCSK1) or type 3 (PCSK3) [10].

As a crucial incretin, GLP-1 is secreted mainly by intestinal epithelial endocrine L cells [9]. The secretion of GLP-1 is closely related to food intake. Eating a meal causes an increase in the secretory activity of L cells in the intestines. The extent of this response after a meal depends on its size and the rate of stomach emptying. The larger the meal size and the faster the stomach empties, the greater the level of glucagon-like peptide-1 [11]. GLP-1 takes part in regulating glucose levels in the bloodstream – it encourages the release of insulin from beta cells in the pancreas and reduces the release of glucagon [9].

This gastrointestinal peptide binds specifically to glucagon-like peptide-1 receptors (GLP-1Rs), which belong to the G-protein-coupled glucagon receptor family [9]. These receptors are located in the lungs, pancreatic islets, heart, vascular smooth cells, macrophages, endothelial cells, central nervous system, kidney, peripheral chemoreceptors (such as the carotid body), and the digestive tract. Furthermore, GLP-1 is able to cross the blood-brain barrier and directly exert influence on the physiological functions of the human brain [12].

GLP-1Rs is present in many tissues, suggesting that their widespread physiological functions beyond glycemic control could potentially include, for example, neuroprotective, anti-inflammatory, and cardioprotective activity [13]. Additionally, glucagon-like peptide-1 has an impact on the digestive tract – by inhibiting the motor activity of the stomach and the secretion of gastric acid. Additionally, it increases satiety and reduces appetite [11].

**Fig. 1:**
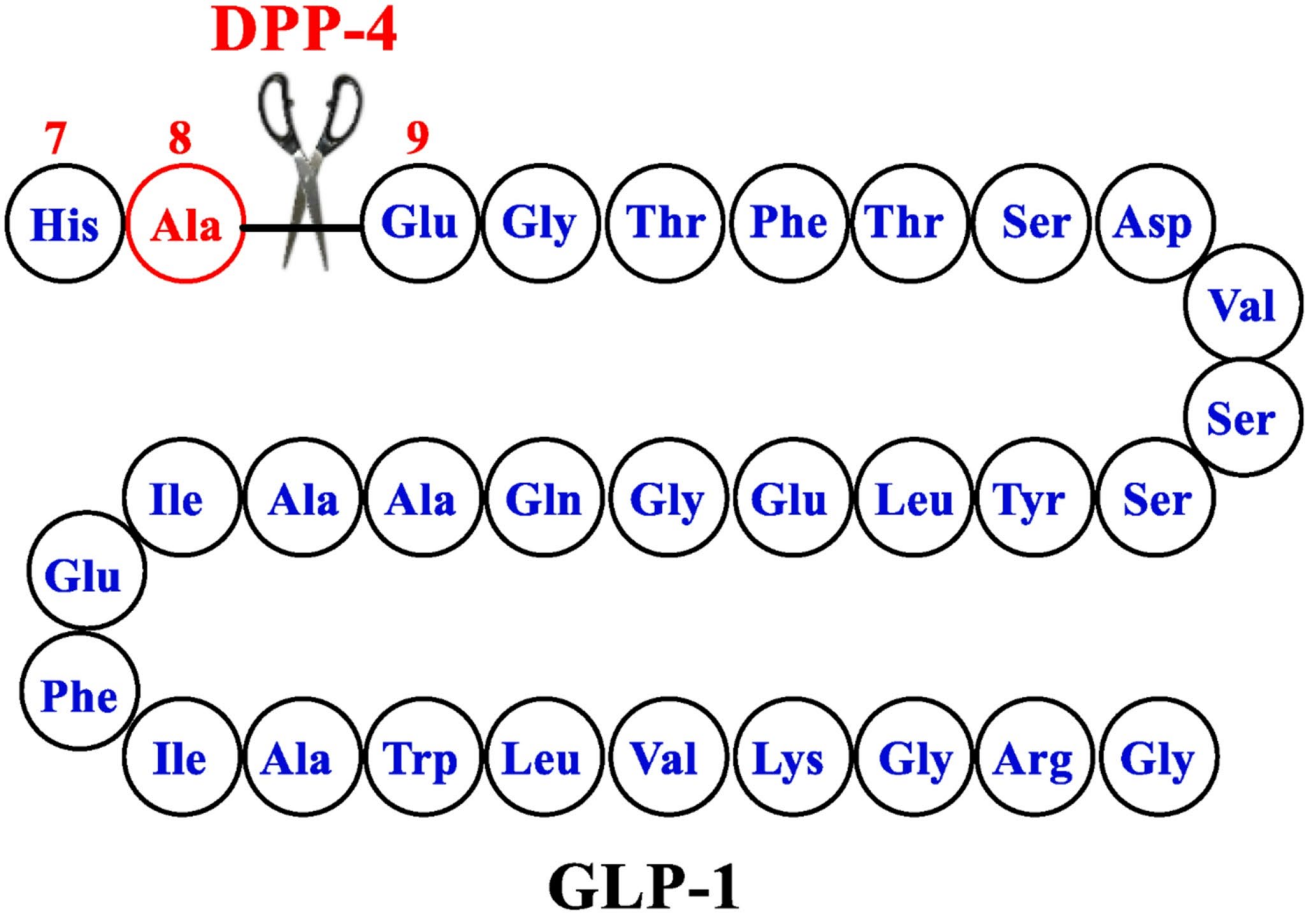
The structure of GLP-1-parent [14]

In the human body, physiological GLP-1 is rapidly metabolized by dipeptidyl peptidase-4 (DPP-4), resulting in a loss of its activity. Its half-life is approximately 2 min. Moreover, GLP-1 is quickly cleared by the kidneys due to high hydrophilicity [14]. The structure of native GLP-1 is presented in Fig. 1.

The pharmacokinetic profile of GLP-1 can be improved in two ways. The first strategy is by inhibiting the activity of the dipeptidyl peptidase-4 by DPP-4 inhibitors. The DPP-4 inhibitors extend the half-life of GLP-1. The second strategy aims to use GLP-1 receptor agonists (GLP-1RAs), also known as GLP-1 mimetics [15].

### Groups of drugs used to treat diabetes

- DPP-4 inhibitor

The DPP 4 inhibitors are categorized into several classes based on the binding of inhibitors to the subsites of DPP-4 protease [16]:

- First class: sitagliptyna, teneligliptyna (more active than sitagliptyna).
  They majority bind to S1 and S2 subsites. Cyannopyrrolidine moiety interacts with S1, whereas hydroxy adamantyl interacts with the S2 subsite.
- Second class: vildagliptin, saxagliptin (more active than vildagliptin).
  These molecules only interact with the S1 and S2 subsites.
- Third class: alogliptin, linagliptin (more active than alogliptin).
  Alogliptin binds to the S1, S2 and S1′ subsites, while linagliptin binds to the same subsites in addition to S2′ [16]. The chemical structures of some DPP4 inhibitors available on the market are presented in Fig. 2.
  **Fig. 2:**
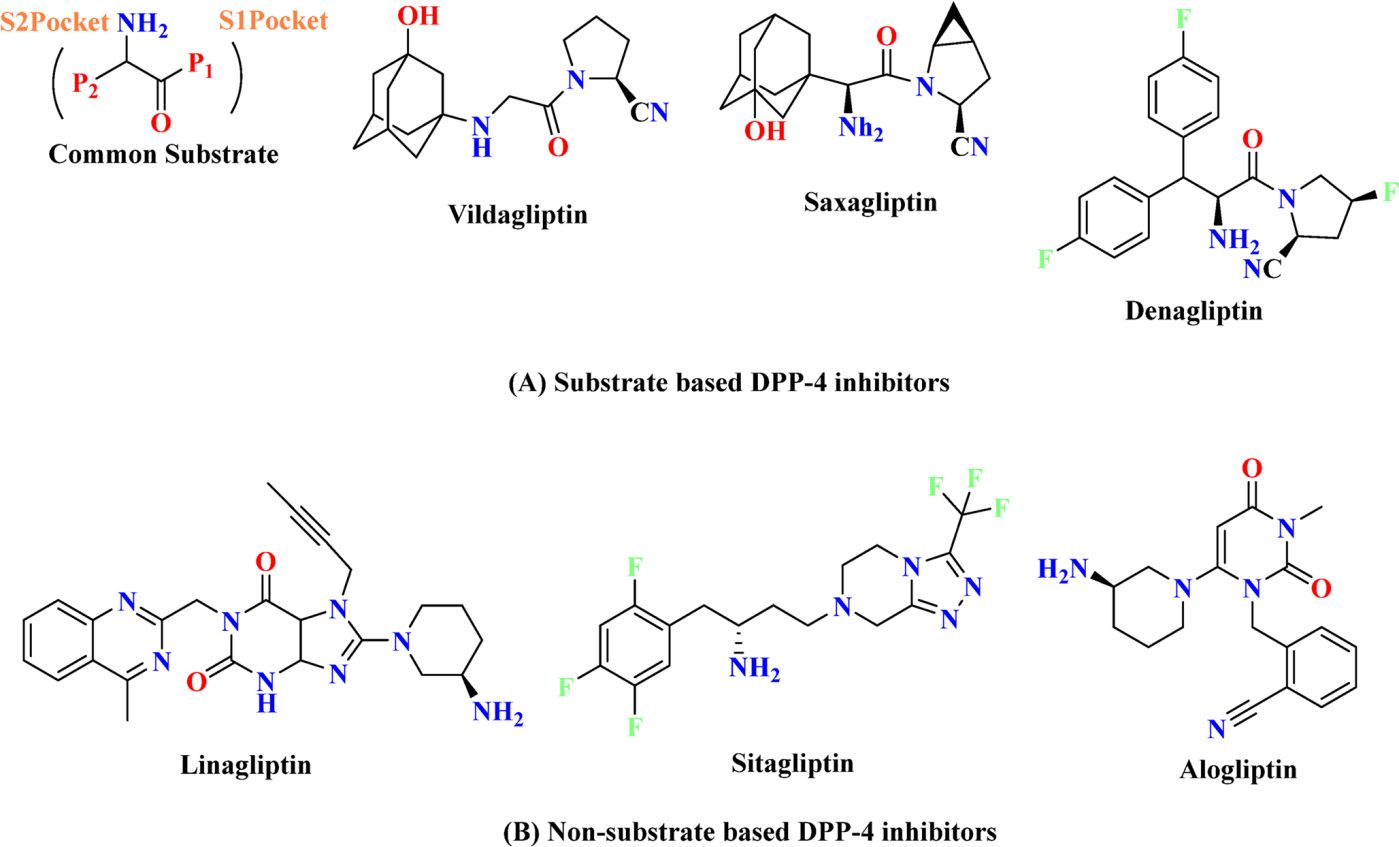
Substrate and non-substrate based DPP-4 market approved inhibitors [16]

**Fig. 3:**
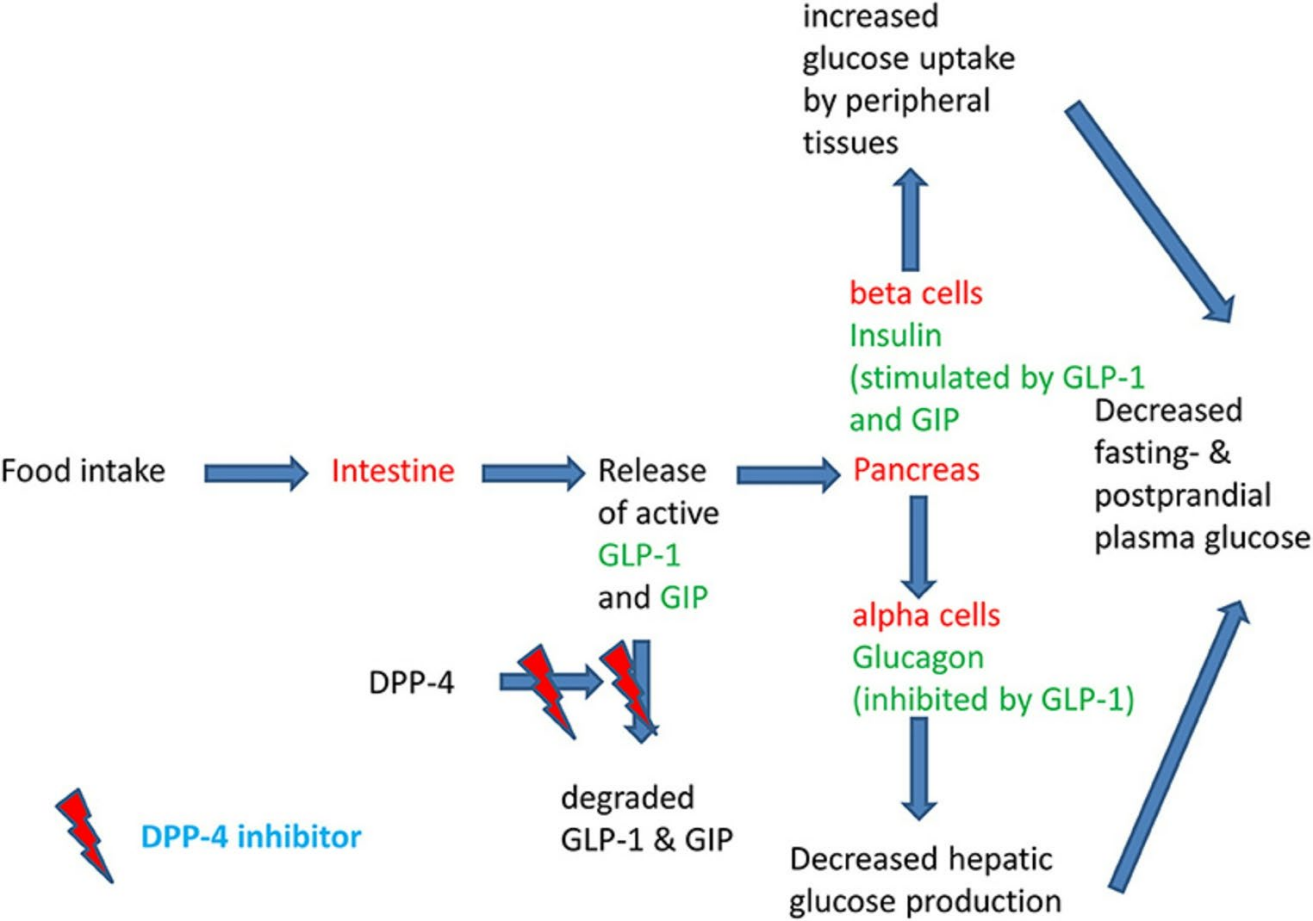
Physiology of the post-prandial regulation of glucose homoeostasis by the incretin system and the action of DPP-4 inhibitors [18]

Their mechanism of action is based on the inhibition activity of dipeptidyl peptidase–4. DPP-4 is responsible for the inactivation of GLP-1 by cleaving two N-terminal amino acids – proline or alanine – from natural peptides in the penultimate (P1) position. GLP-1 is responsible for the increased secretion of insulin from pancreatic β-cells. It also acts upon α-cells, leading to the suppression of glucagon release. This mechanism contributes to effective glucose control in the human bloodstream [17]. Figure 3 shows a scheme of the physiology of the incretin hormones after a meal and the mode of action of DPP-4 inhibitors [18].

DPP-4 inhibitors as oral drugs also exhibit other valuable properties. They can reduce inflammation and impact the cardiovascular system – improving indicators such as: PFR (indicator of diastolic function) and LVEF (an indicator of left ventricular systolic dysfunction) [19]. Moreover, sitagliptin, for example, could be a promising potential agent against the development of Parkinson’s disease [20].

DPP-4 inhibitors can also cause several side effects, such as headache, nasopharyngitis, nausea, arterial hypertension, heart failure, pancreatitis and inflammatory bowel disease, and various skin reactions (pruritus, bullous pemphigoid, rash, angioedema and photosensitivity). Bullous pemphigoid is an autoimmune disorder that occurs in older people, characterized by widespread pruritic tense vesicles and bullae on the skin. Moreover, sitagliptin, linagliptin, saxagliptin, and alogliptin could induce joint pain [20, 21].

- GLP-1 agonists

GLP-1 receptor agonists are categorized in two groups based on their duration of action [22]:

-  short-acting GLP-1RAs: lixisenatide, exenatide (twice daily).
-  long-acting GLP-1RAs: liraglutide, dulaglutide, semaglutide (once-weekly injections or once-daily oral administration). Oral semaglutide has the longest half-life (165–185 h) of the long-acting GLP-1RAs.

**Fig. 4:**
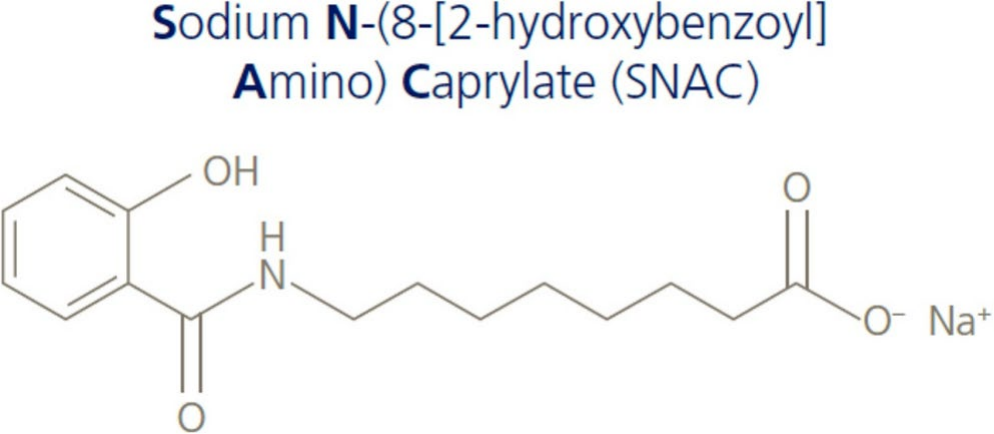
Structure of sodium N-(8-(2-hydroxybenzoyl) amino) caprylate [23]

To extend the half-life of long-acting GLP-lRAs, various modifications have been made. Exenatide is encapsulated in a dissolvable microsphere. Semaglutide (injectable form) and liraglutide have been modified by a fatty acid chain that aids reversible binding to albumin. The fatty acid chain that has been attached to dulaglutide facilitates covalent binding to carrier molecules such as domains of immunoglobulin G. Oral semaglutide is a combination of semaglutide and the absorption enhancer sodium N-(8-(2-hydroxybenzoyl)amino) caprylate (SNAC) (Fig. 4) [23]. SNAC prevents the degradation of semaglutide by gastric acid and facilitates the absorption of semaglutide through the gastric mucosa to systemic circulation by increasing pH in the stomach [24, 25].

GLP-1 agonists induce the direct activation of the GLP-1 receptor, leading to the stimulation of pancreatic insulin secretion and the inhibition of glucagon secretion [26]. These are primarily used in the treatment of type 2 diabetes mellitus. Indications for GLP-1 agonist therapy include contraindications or intolerance to metformin or poorly controlled diabetes in patients already taking metformin. Furthermore, liraglutide and semaglutide are prescribed as drugs for obesity or overweight [27].

## Properties and applications of semaglutide

The primary indication of semaglutide is type 2 diabetes. Currently, it is used as medication for adults who are obese or overweight, provided they have at least one weight-related comorbidity (for instance, high blood pressure, type 2 diabetes, or high cholesterol) [28].

Semaglutide, as a glucagon-like peptide-1 receptor agonist, increases the secretion of insulin from pancreatic β-cells. Addictionally, it supress glucagon release from pancreatic α-cells. It also contributes to a reduction in hunger and food cravings, and stimulates the satiety center in the hypothalamus, thereby increasing a feeling of fullness. It causes weight loss by reduced energy intake with minimal effects on energy expenditure.

**Fig. 5:**
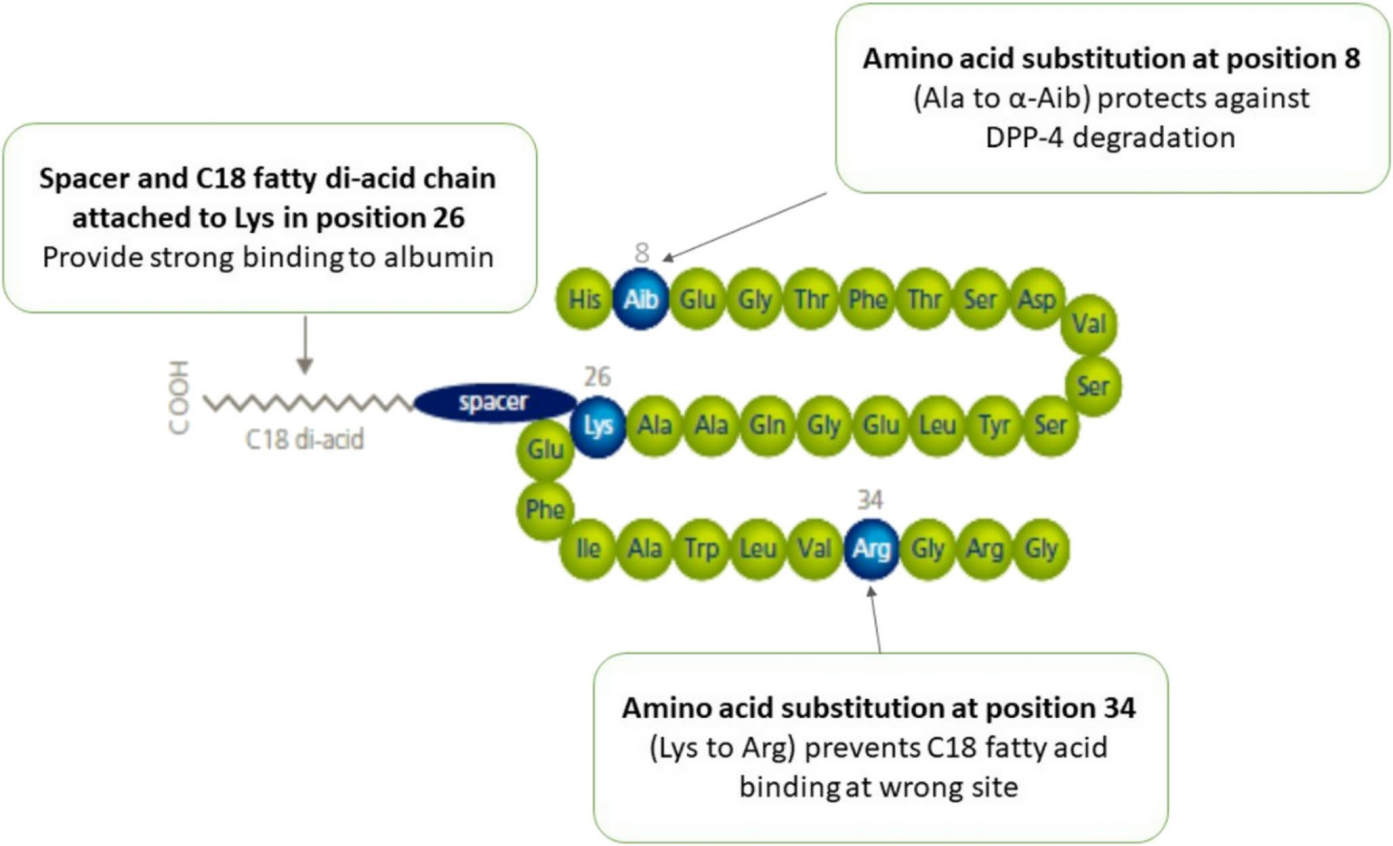
Structure of semaglutide (subcutaneous formulation) [23]

Semaglutide is available in two forms: oral and injectable (under the skin fold). The structure of semaglutide in the subcutaneous formulation is presented in Fig. 5 [23]. Oral tablets of Ozempic are often administered once a day at doses of 7 mg and 14 mg, with an initial dose of 3 mg once a day for the first 30 days [25]. Oral absorption of semaglutide is considerably faster than subcutaneous administration. However, other oral drugs should be administrated 30 min after swallowing the semaglutide tablet as they can disturb the absorption process. The elimination of this medication is very similar for both the oral and injectable form [25, 29].

The SURPASS-2 trial compared the efficacy and safety of tirzepatide at doses of 5 mg, 10 mg, and 15 mg, with those of semaglutide at a dose of 1 mg in patients with type 2 Diabetes over a period of 40 weeks [30]. The overall mean duration of Diabetes was 8.6 years, the average body weight was 93.7 kg, and the mean glycated hemoglobin level was 8.28%. The final and mean points of reduction in body weight show that tirzepatide at doses of 5 mg, 10 mg, and 15 mg were − 7.6 kg, − 9.3 kg, and − 11.2 kg, respectively, compared to −5.7 kg for semaglutide at a dose of 1 mg. Both drugs also decreased glycated haemoglobin levels. The reductions observed in glycated haemoglobin levels with tirzepatide at a dose of 5 mg, 10 mg, and 15 mg were − 2.01% points, − 2.24% points, and − 2.30% points, respectively, compared to − 1.86% points with semaglutide at a dose of 1 mg. These final results confirm the primary pharmacological effects of semaglutide.

**Fig. 6:**
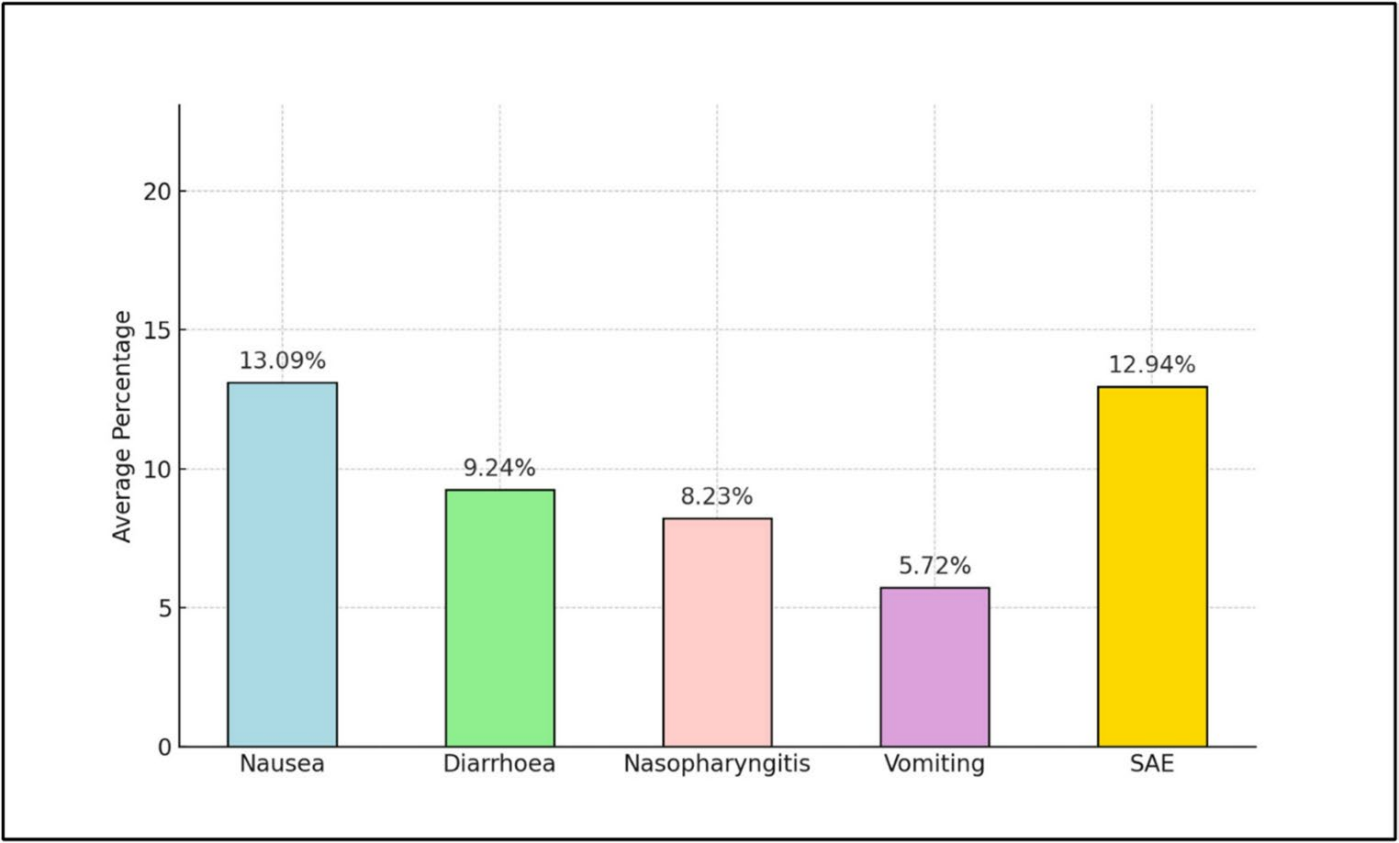
Aggregate mean values with adverse events and complications across all studies after semaglutide use [28]

However, Ozempic does not exhibit greater impact on gastric emptying [31]. Semaglutide may also cause some side effects, the frequency of which is shown in Fig. 6 [28].

Ozempic is available in injectable form. In the treatment of type 2 Diabetes, Ozempic is administered subcutaneously once a week at doses of 0.5 mg and 1.0 mg, with an initial dose of 0.25 mg/week for the first 4 weeks [25]. However, in the treatment of obesity, this drug is used in larger doses – 2.4 mg/week [32].

## Safety of semaglutide treatment

### Risk of thyroid cancer

According to recent research from the GAROS study, semaglutide may have an impact on thyroid hormones, such as free triiodothyronine (fT3), free thyroxine (fT4), and thyroid-stimulating hormone (TSH). The study involved obese patients without diabetes [33]. After three months of semaglutide treatment, levels of TSH and fT3 were decreased. However the level of fT4 increased to approximately 0.06 ng/dl. Upon discontinuation, TSH returned to its baseline value; however, fT3 and fT4 levels did not fully return to their original values. Notably, changes in fT4 were negatively associated with weight change during and after treatment with semagutide – unlike TSH and fT3.

However, according to these studies, the incidence of thyroid cancer in patients taking semaglutide was less than 1%, suggesting no significant risk.

Chao et al. reported that semaglutide is contraindicated in patients with a personal or family history of medullary thyroid cancer [34].

Other studies have also shown that semaglutide was not associated with an increased risk of any types of cancer [28].

### Cardiovascular system

A recent study has indicated that semaglutide might inhibit the expression of S100a8, S100a9, and Cxcl2 in cardiac neutrophils in the context of obesity. The study was conducted on C57bl6 mice [35]. They were divided into two groups: the first group was fed a standard chow diet, and the second group received a high-fat diet. After 12 weeks, mice in the high-fat diet group were divided into two groups: the first group included the high-fat group, which continued to eat a high-fat diet; and a second group, or the semaglutide intervention group, in which mice were fed a high-fat diet and treated with semaglutide at 30 nmol/kg/day, injected intraperitoneally for 12 weeks. The decrease in S100a8, S100a9, and Cxcl2 levels suppress inflammation and oxidative stress in cardiac muscle, thereby reducing the risk of myocardial infarction, heart failure, arteriosclerosis, and myocardial reperfusion injury.

Pan et al. reported that semaglutide inhibits the expression of miR-155 in exosomes derived from the Raw264.7 macrophage cell line [36]. MiR-155, as microRNA, impairs the function of endothelial progenitor cells and induces inflammatory processes in macrophage-derived exosomes. These anti-inflammatory effects of semaglutide decreases cardiovascular risk and boosts cardiac function. Furthermore, semaglutide has been shown to improve other protective functions such as the migratory, tube-forming, and proliferative capacities of endothelial progenitor cells.

Additionally, semaglutide modulates the release of exosomes by epicardial fat, reduces neutrophil (dHL60) adhesion to endothelial cells, and enhances the angiogenesis process [37].

One recent study demonstrated that semaglutide inhibits neutrophil activation in epicardial fat and their adhesion to endothelial cells [38]. In addition, semaglutide suppresses the expression of miR-155. MiR-155, as microRNA, inducesinflammation in macrophage-dervied exosomes. These anti-inflammatory effects of semaglutide decrease cardiovascular risk and boost cardiac function.

Semaglutide may also reduce swelling of aortic endothelial cells and prevent their detachment from the internal elastic lamina, thereby lowering the risk of atherosclerosis. Moreover, it boosts endothelial permeability by reducing the levels of Coll5a1, Lama4, and Sparc in the extracellular matrix and cytoskeleton [39].

Semaglutide also mitigates symptoms of heart failure [40] and improves the angiogenesis process [37]. On the other hand, semaglutide increases heart rate by 1 to 4 beats per minute, so regular monitoring of pulse rate is very important [31].

### Inflammation

Semaglutide also exhibits anti-inflammatory properties, as it modulates or reduces inflammatory processes. It may inhibit the release of pro-inflammatory cytokines, such as IL-6 and TNF-α.

Research conducted on H9c2 embryonic rat heart-derived cells indicated that semaglutide could play a role in inhibiting the occurrence of inflammatory responses by suppressing the expression of proteins such as NF-κB, TNF-α and IL-1β [41]. Chronic administration of semaglutide (dose = 0.9 mg/kg) in the research sample contributed to a significant reduction in the concentration of the above proteins associated with inflammation.

### Neuroprotective activity

Another study deomonstrated the neuroprotective properties of semaglutide [42]. This glucagon-like peptide-1 receptor agonist reduced the level of TLR4\STAT3 in the brain tissue of mice during endotoxemia and polymicrobial sepsis.

Furthermore, in research involving the brain tissues of rats, semaglutide was also found to reduce inflammation signaling pathways, including p38 MAPK, c-Jun, and Nf-κB p65. These actions of semaglutide are likely responsible for its neuroprotective effects and its ability to enhance cognitive function [38].

### Impact of pancreas

Patients with obesity might also have damaged pancreatic islets. Research investigating the impact of semaglutide on islet structural remodeling and its endocrine function in diet-induced obese mice demonstrated that the glucagon-like peptide-1 receptor agonist increased islet cell proliferation and restored islet size and alpha- and beta-cell masses [43]. It reduced pro-inflammatory markers and improved the Pancreatic duodenal homeobox 1, glucose transporter 2, and peroxisome proliferator-activated receptors alfa and gamma.

### Vision

Long-term elevated blood sugar levels can lead to diabetic retinopathy, a complication characterized by extensive damage to the retina’s blood vessels, resulting in microaneurysms, hemorrhages, and macular edema, potenitally even leading to vision loss. Recent concerns have arisen as to the potential association between semaglutide and retinopathy progression in diabetics.

Hathaway et al. reported that semaglutide may contribute to a higher risk of intensity of retinopathy (particulary in diabetics with existing retinopathy), with a rapid reduction in hemoglobin A1c levels and a higher rate of progression of proliferative retinopathy, as well as risk of new-onset macular edema [44].

Another three-year clinical study involved 87 patients with at least 1 year of semagluide use, complete documentation of retinopathy levels, visual acuity, and central subfield thickness [45]. During the study period, 63.2% of patients required intravitreal anti-vascular endothelial growth factor injections. Visual acuity remained constant for 72.4% of patients, with 16.1% experiencing vision loss and 11.5% showing improvement. The study did not reveal a clear association between semaglutide treatment and an increased risk of progression of diabetic retinopathy or vision loss. Therefore, further research is needed to draw conslusive evidence.

### Mood disorder

According to research investigating the relationship between semaglitude treatment for diabetes/obesity and mood disorders, semaglutide did not exhibit a negative impact on mood [46, 47]. Patients with obesity did not complain about worsening of mood or suicidal thoughts. Furthermore, they were pleased with losing weight due to treatment with this glucagon-like peptide-1 receptor agonist.

However, several cases have been reported, including the case of a woman with type 2 diabetes, hypertension, hyperlipidemia, and depression [48]. After four weeks of treatment with semaglutide, she became more irritable and anxious, experienced a loss of interest in activities that she previously enjoyed, and had difficulty falling asleep. These long-term symptoms are dangerous and risky, as they may potentially even lead to depression. After the discontinuation of semaglutide, her depressive mood resolved.

### Fertility

Women with obesity and/or diabetes sometimes struggle with polycystic ovary syndrome (PCOS), a hormonal imbalance that occurs due to excessive androgen production by the ovaries. This disorder contributes to infertility, weight gain, insulin resistance, and abnormal heart function. Recent research shows that semaglutide aids not only in weight reduction in PCOS patients but also impacts the reproductive system. Semaglutide increases fertility by helping toreduce inflammation and fibrosis in the ovaries [49].

### Side effects

Semaglutide may also cause various side effects, the frequency of which is shown in the Fig. 6 [28].

The most common side effects of using semaglutide are related to the digestive system. Nausea, diarrhea, and vomiting are reported most frequently. Gastrointestinal problems increase with higher doses [28].

Other adverse events include nasopharyngitis, hypoglycemia, increased lipase levels, lack of appetite, gastroparesis, bowel obstruction, abdominal pain, dyspepsia, constipation, influenza symptoms, and headaches [28].

Semaglutide can also cause skin problems. Pruritus is the most common symptom of an injection site reaction. Fortunately, it is transient and patients do not require the cessation of therapy [50].

Pancreatic inflammation, including hemorrhagic and necrotizing pancreatitis is a rare but very serious potential side effect of using semaglutide. This drug stimulates pancreatic islet beta cells and exocrine duct cells leading to ductal overgrowth and narrowing, which increases pancreatic mass and the risk of ductal occlusion. This may predispose individuals to pancreatitis [50].

Moreover, the appearance of the so-called “Ozempic face” is an increasingly common side effect. “Ozempic face” is related to rapid weight loss, particularly to a significant loss of fat in the face. Ozempic users complain about wrinkles, sunken eyes, a hollowed appearance, sagging jowls around the neck and jaw, and alterations in the cheeks, lips, and chin [51].

### Contraindication

Semaglutide is contraindicated in pregnancy. Patients (female and male) should not take semaglutide at least 2 months before a planned pregnancy due to a concern for potential fetal damage [52].

Contraindications also apply to patients with a history of medullary thyroid cancer, multiple endocrine neoplasia syndrome type 2, and hypersensitivity to semaglutide [31].

## Pharmacokinetics

In recent years, a various studies have investigated the pharmacokinetics of semaglutide, including pharmacokinetics in healthy individuals, pharmacokinetics in patients with various diseases, and the effects of drug–drug interactions and drug–food interactions on the pharmacokinetics of semaglutide in humans.

The safety and pharmacokinetics of oral semaglutide were investigated in two randomized, double-blind, placebo-controlled trials [53]. The pharmacokinetic properties of oral semaglutide were similar in healthy subjects and subjects with type 2 diabetes. The elimination half-life (t½) of semaglutide was comparable between healthy subjects and subjects with type 2 Diabetes, with geometric means of 153, 161 and 158 h in healthy subjects receiving 20 mg and 40 mg doses and subjects with type 2 Diabetes receiving 40 mg, respectively. Semaglutide was metabolized through proteolytic cleavage of the peptide backbone and sequential β-oxidation of the fatty acid side chain.

Many reports indicated that semaglutide exhibits a predictable pharmacokinetic profile, supporting its Longer duration of action allowing for once-weekly subcutaneous administration. The subcutaneous bioavailability of semaglutide was determined as approximately 94% [54]. Maximum concentration of the drug was observed after 33–36 h, after the administration of a1.0 mg dose [54]. The volume of distribution was reported at 0.102 L/kg, with a body clearance of 0.0016 L/h/kg for semaglutide [55]. Jensen et al. reported that semaglutide was metabolized into six different metabolites, identified as P1–P3 and P5–P7. P4 represents the parent compound [3 H]-semaglutide, which was the primary component detected in plasma [56]. The authors reported that semaglutide is metabolized via proteolytic cleavage of the peptide backbone and b-oxidation of the fatty acid side chain. The mean total recovery of semaglutide and its metabolites was 75.1%, with 53% in urine and 18.6% in faeces, and a minor amount (3.2%) in expired air [56].

Kim et al. conducted researchto characterize the pharmacokinetics of semaglutide in different animal species and to develop a pharmacokinetic model incorporating allometric scaling for human pharmacokinetics prediction [57]. Pharmacokinetic data were obtained following intravenous injection of semaglutide in mice, rats, and dogs. The drug’s pharmacokinetics were characterized through interspecies scaling, and human pharmacokinetics were predicted using a population pharmacokinetic model integrating allometric scaling. Based on the developed model, it was estimated that for humans, the median clearance was 0.0397 L/h, with a volume of distribution of 6.23 L.

Patients with type 2 diabetes often have comorbidities, such as cardiovascular disease, hepatic disease, or chronic kidney disease; therefore, it is essential to study the influence of these conditions on semaglutide treatment. The pharmacokinetics and tolerability of semaglutide were investigated in patients with and without renal impairment [58]. Semaglutide exposure in patients with mild and moderate renal impairment was similar to that in patientswith normal renal function. However, in patients with severe renal impairment, a 22% higher mean exposure was observed compared to patients with normal renal function. The oresults indicated that semaglutide appears to be a useful treatment option for patients with type 2 diabetes who also have impaired renal function, including patients undergoing hemodialysis.

The pharmacokinetics and tolerability of semaglutide in individuals with hepatic impairment were investigated using the Child–Pugh criteria [59]. The authors concluded that the pharmacokinetic properties of semaglutide in patients with hepatic impairment were similar to those of patients with normal hepatic function. Bækdal et al., based on pharmacokinetic investigations, did not observe any hepatic impairment, regardless of severity, on the pharmacokinetics of orally administered semaglutide [60]. These results suggest that dose adjustment in patients with hepatic impairment may not be necessary.

Further investigations indicated that the age of patients with type 2 diabetes does not affect the glycemic efficacy of oral semaglutide and the presence of upper gastrointestinal disease, or that hepatic impairment did not affect the pharmacokinetics of semaglutide [61]. Overgaard et al. also reported that gender, age, renal impairment, and hemodialysis had no significant effect on semaglutide action [62]. The authors compared the availability of pharmacokinetic data from patients treated with oral versus injectable semaglutide, and thus provided the opportunity to compare clinically meaningful outcomes achieved with different routes of drug administration. Very simillar exposure-response pharmacodynamic relationships were demonstrated based on plasma semaglutide levels achieved through both oral and injectable administration.

Jensen et al. investigated the absorption, metabolism, and excretion of a single 0.5 mg/450 µCi [16.7 MBq] subcutaneous dose of [3 H]-radiolabelled semaglutide in healthy human subjects and compared the results with data from nonclinical studies [56]. The results indicated that semaglutide was metabolized through proteolytic cleavage of the peptide backbone and sequential beta-oxidation of the fatty acid sidechain. Intact semaglutide was the primary circulating component in plasma (69–83% of the total amount of semaglutide-related material). Semaglutide was excreted mainly in urine and to a lesser extent in feces. The median t_max_ after administration of a single dose of 0.5 mg subcutaneous semaglutide was 56 h, the geometric mean half-life (t_1/2_) was 168 h, and the AUC_0–∞_ was 3123.4 nmol ∗ h/L. Similar investigations were also performed on animals (rats and monkeys), with no significant differences observed between species.

Overgaard et al. investigated the absorption, distribution, and elimination of semaglutide by means of population pharmacokinetic models using data from clinical pharmacology trials conducted in patients with type 2 diabetes and healthy subjects [63]. Data were obtained from trials with subcutaneous and intravenous administration of semaglutide. The effects of various dosing conditions – e.g., fasting time and water volume, demographic covariates, and variability in exposure – following single and multiple dose oral administration were investigated. Semaglutide demonstrated similar pharmacokinetics in healthy individuals and patients with type 2 diabetes following oral, subcutaneous and intravenous administration. The bioavailability and exposure of oral semaglutide were reduced by a shorter post-dose fasting time (15 min) and the co-administration of a larger volume of water (240 mL).

The effect of various drugs on the pharmacokinetics of semaglutide was also investigated. For example, a single-center, randomized, open-label, parallel-group trial investigated pharmacokinetic interactions of oral semaglutide with omeprazole in 54 healthy subjects [64]. A slight, but non-statistically significant, increase in semaglutide exposure when oral semaglutide was administered with omeprazole was observed. The authors concluded that the observed increase in drug exposure is not clinically significant and likely does not require dose adjustment.

Some recent studies have indicated that semaglutide also acts on the brain, suggesting its potential usefulness in the treatment of various diseases, including neurodegenerative diseases such as Alzheimer’s and Parkinson’s diseases [65]. For this reason, Lee et al. analyzed semaglutide in rat plasma and brain to characterize the pharmacokinetics and brain distribution of the drug. After subcutaneous injection, the plasma concentration of semaglutide gradually increased, reaching maximum concentrations in 3–12 h, and then decreased with an average t_1/2_ of 7.22–8.99 h, with peak concentrations of 24,943.33 ± 1087.19 and 27,636.5 ± 2331.98 ng/mL, respectively. The total brain concentration of semaglutide was not very high, with the average partition coefficient of < 0.0005. However, significantly higher concentrations of semaglutide in the rat hypothalamus than those in other parts of brain were observed. The average concentration in the hypothalamus was 2.26-fold higher than those in the total brain. The partition coefficient.

of semaglutide was also 1.82-fold higher than those in the total brain. These results indicate that semaglutide is specifically distributed to the hypothalamus, a brain region that is not protected by the blood–brain barrier. This targeted distribution may help to understand the drug’s action in the brain.

A powerful strategy for treating type 2 diabetes is combination therapy with basal insulin and semaglutide to harness their different mechanisms and target the various tissues involved in glucose regulation. The pharmacokinetics of a once-weekly fixed-ratio combination of insulin icodec and semaglutide was investigated [66]. In the study, 31 patients with type 2 Diabetes received single subcutaneous injections of IcoSema, icodec, or semaglutide, with 6–9 weeks’ washout between doses. Pharmacokinetic blood samples were taken up to 840 h post-dose. Results indicated that the pharmacokinetics of both icodec and semaglutide were unaffected by the combination of icodec and semaglutide in IcoSema (the combination of the once-weekly basal insulin icodec and the once-weekly glucagon-like peptide-1 receptor agonist semaglutide). However, the maximum concentration of semaglutide was higher and occurred earlier, likely due to icodec outcompeting semaglutide for albumin binding locally at the injection site.

Nielsen et al. conducted pharmacokinetic studies to confirm bioequivalence between 2G and 1Goral semaglutide formulations at steady state in healthy participants [67]. The authors found that the 2G oral semaglutide formulation is bioequivalent to 1G oral semaglutide, with no new safety concerns identified.

The effects of various dosing schedules on the pharmacokinetics of oral semaglutide was investigated [68]. The objective of the the study was to explore whether alternative dosing schedules of oral semaglutide during the day or evening, with different pre- and post-dose fasting times, could provide similar exposure compared to dosing on an empty stomach. Results indicated that shorter pre-dose fasting times compared with an overnight pre-dose fast resulted in significantly lower semaglutide AUC_0–24 h_ and C_max_ after the 10th dose.

The pharmacokinetics of a once-a-month single-dose semaglutide microcapsule were studied. This formulation effectively suppressed the initial burst release down to ⁓10%, and maintained therapeutic plasma drug concentrations for a month [69].

The absorption, distribution, and elimination of oral semaglutide were investigated using a population pharmacokinetic model based on data from clinical pharmacology trials [63]. The study examined the effects of various dosing conditions (fasting time and water volume), demographic covariates, and variability in exposure following single and multiple oral administrations. Bioavailability in subjects with type 2 diabetes was also investigated in a separate analysis. Lower bioavailability and exposure of oral semaglutide were observed after shorter fasting time before treatment (15 min) and the co-administration of a larger water volume (240 mL). The within-individual variation from dose to dose in oral semaglutide bioavailability was high, but, as a result of daily dosing and a long plasma half-life (1 week), the variability of semaglutide exposure decreased considerably at a steady state.

The absorption, distribution, and elimination of semaglutide by means of population pharmacokinetic models using data from nine clinical pharmacology trials conducted in both healthy subjects and those with type 2 diabetes was also investigated [70]. In a typical subject with type 2 Diabetes, clearance was estimated at 0.0348 L/h, and the central and peripheral volumes were estimated at 3.59 L. Peak semaglutide concentrations were reached within tree days of starting treatment. However, steady-state levels were achieved after 4 to 5 weeks of weekly administration.

In other investigations based on the population pharmacokinetic model, no clinically significant effects of exposure on semaglutide were observed based on gender, age, race, ethnicity, renal function, or injection site [71]. Only the patient’s body weight was found to have an effect on semaglutide exposure. Higher body weight caused a proportional decrease in drug exposure.

Based on the conducted studies, general conclusions can be drawn that semaglutide has a predictable pharmacokinetic profile with a long t1/2, allowing for once-weekly subcutaneous administration. Oral absorption of semaglutide is significantly faster than subcutaneous administration, while the elimination of subcutaneous and oral semaglutide is similar. Additionally, food intake and various dosing conditions, including water volume and dosing schedules, can affect the exposure of oral semaglutide. The half-life of semaglutide was comparable between healthy subjects and individuals with type 2 diabetes.

## Analysis of semaglutide in various samples by chromatography

The use of semaglutide has increased worldwide in recent years due to its effectiveness in treating type 2 diabetes. Owing to its appetite-regulating effects, it is also used in many countries to treat obesity. However, semaglutide is also often misused by non-diabetic and non-obese individuals. Consequently, there is a growing need to develop procedures for the analysis of this drug. In the literature, there is a limited number of published procedures for the quantification of semaglutide in various biological samples. Several studies have also been performed on the pharmacokinetics of semaglutide by chromatography, most often using LC MS/MS.

**Fig. 7:**
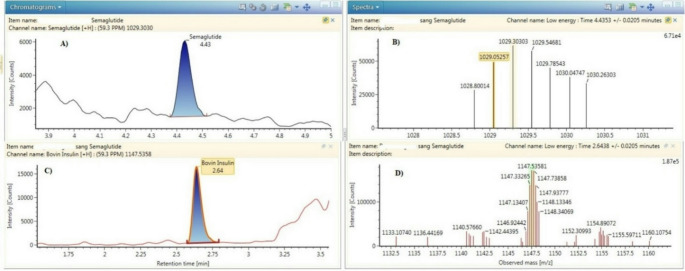
Chromatograms and spectra obtained after the extraction of subject’s B blood sample. The concentration measured was 36 ng/mL. Image A and B show semaglutide chromatogram and spectrum obtained at low collision energy. Images C and D show insulin bovine (I.S.) chromatogram and spectrum obtained at low collision energy [73]

A liquid chromatography coupled with high-resolution mass spectrometry (LC-HRMS) procedure was developed and validated for the identification and quantification of semaglutide in human whole blood [72]. In the procedure, bovine insulin was used as an internal standard and added to blood samples, followed by protein precipitation using a mixture of acetonitrile and methanol (70:30,v: v). Chromatographic separation was performed on a Waters Acquity CORTECS C18 + column using a mixture of acetonitrile and water acidified with 0.1% formic acid. Figures 7A and B show the chromatogram and mass spectrum of semaglutide extracted from patients’ blood samples at low collision energy, while Figs. 7C and D show the chromatogram and spectrum of bovine insulin obtained at low collision energy [72]. The limit of quantification (LOQ) obtained by the procedure was 2 ng/mL, which represents one-third of the residual concentration following administration of the lowest therapeutic dose of semaglutide.

Since the neuroprotective effect of semaglutide in neurodegenerative diseases has been reported, there is also a need to develop procedures for the determination of this drug in brain tissue.

**Fig. 8:**
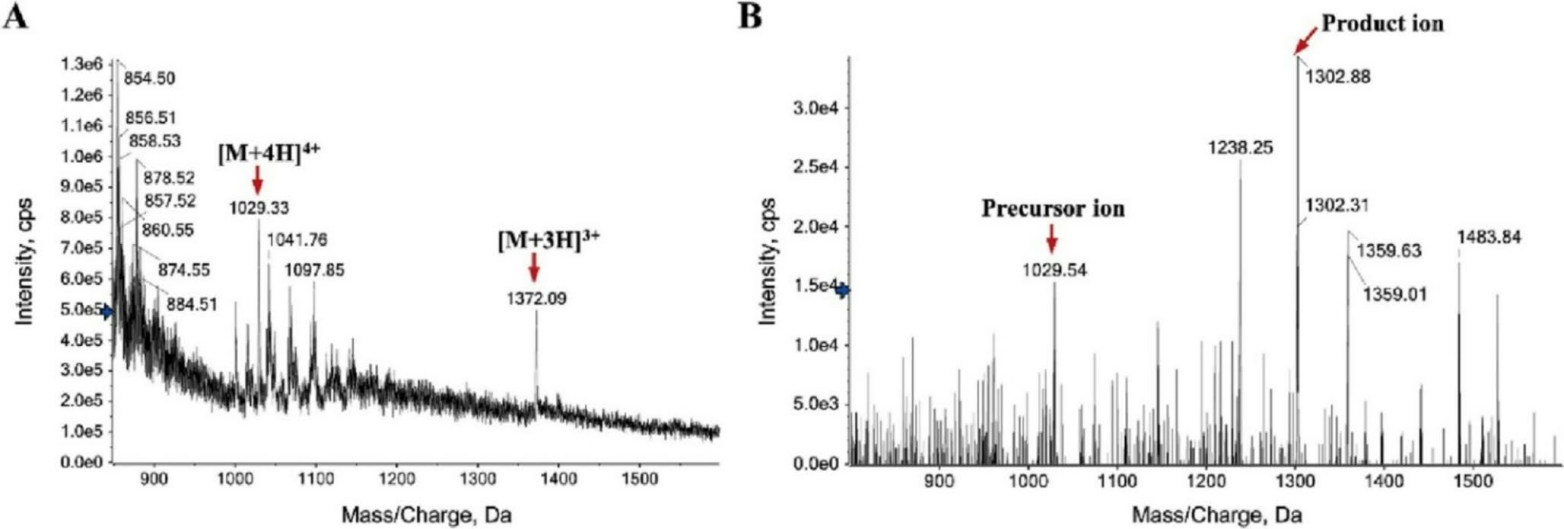
Full Q1 scan (**A**) and product ion (**B**) spectra of semaglutide [74]

A liquid chromatography-tandem mass spectrometry (LC-MS/MS) procedure for the analysis of semaglutide in plasma and brain tissue was developed to characterize the pharmacokinetics and brain distribution in rats [73]. The analysis was performed on a Peptide CSH C18 column with a mobile phase containing 0.1% formic acid in water and 0.1% formic acid in acetonitrile. The condition was applied to in vivo pharmacokinetic investigations. Based on the obtained results, the plasma concentration of semaglutide increased, reached maximum concentrations in 3–12 h, and decreased with an average t1/2 of 7.22–8.99 h. The absolute bioavailability of the drug after injection was 76.65–82.85% in rats. The authors determined significantly higher concentrations of semaglutide in the hypothalamus compared to other parts of the brain, indicating that the drug effectively reaches regions associated with the GLP-1 receptor, which are related to appetite suppression and neuroprotection. Figure 8A shows the full Q1 mass scan spectrum of semaglutide.

A UPLC BEH300 C18 column was used for the analysis of semaglutide in human plasma samples by LC-MS/MS [74]. Figure 8A shows the full Q1 mass scan spectrum of semaglutide. Due to the large molecular weight of semaglutide (m.w. = 4114), the authors observed multiply charged precursor ions in the full scan of protonated semaglutide. Figure 8B shows the product ion mass spectra of the protonated semaglutide with several potential ion fragments. Based on their results, the authors concluded that the overall exposure of baseline-corrected total thyroxine was increased when levothyroxine was co-administered with semaglutide. This indicates that the co-administration of both drugs may reduce semaglutide absorption.

To investigate the effect of omeprazole on the pharmacokinetics of oral semaglutide determination, plasma concentrations of both drugswere determined using LC-MS/MS following plasma protein precipitation [64]. Chromatographic separation was performed using a UPLC BEH300 C18 column. Analytes were detected using a triple quadrupole mass spectrometer operated in negative ion mode.

**Fig. 9:**
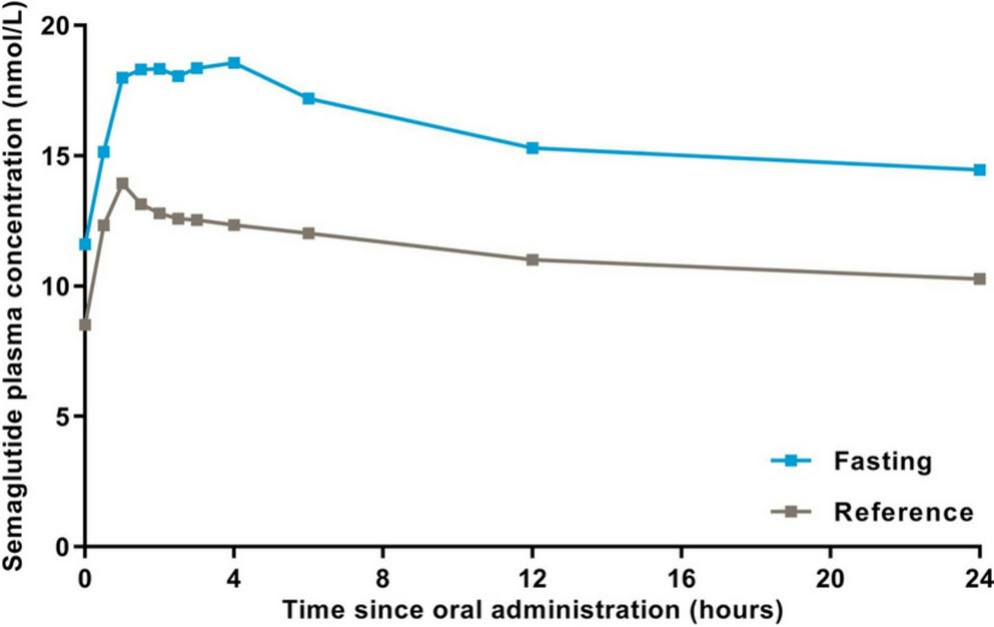
Geometric mean semaglutide plasma concentration–time profiles for fasting and reference treatment arms on day 10 of once-daily dosing of oral semaglutide in healthy subjects (food-effect trial). *n* = 26 per treatment arm [76]

LC-MS/MS was used for the analysis of semaglutide in human plasma on a UPLC BEH300 C18 column to investigate the effects of various dosing schedules on the pharmacokinetics following oral administration of the drug [68]. The same LC-MS/MS conditions were also applied to determine semaglutide concentrations in human plasma during studies evaluating the drug’s absorption, distribution, and elimination after oral administration [63]. The same determination procedure was used in investigations assessing the effect of various dosing conditions on the pharmacokinetics of oral semaglutide [75]. The obtained results, presented as geometric mean semaglutide plasma concentration–time profiles for fasting and reference treatment arms on day 10 of once-daily dosing of oral semaglutide in healthy subjects, are presented in Fig. 9.

Kapitza et al. investigated the influence of semaglutide on the bioavailability of ethinylestradiol or levonorgestrel. The analysis of semaglutide in human plasma samples by LC-MS/MS was performed on a UPLC1 BEH300 C18 column [76]. The drug was detected using a triple quadrupole mass spectrometer with atmospheric pressure ionization monitoring positive ions. The authors reported a lower Limit of quantification for semaglutide of 1.94 nmol/L. The same chromatographic procedure was used to evaluate the pharmacokinetics, safety, and tolerability of once-weekly subcutaneous semaglutide [53].

**Fig. 10:**
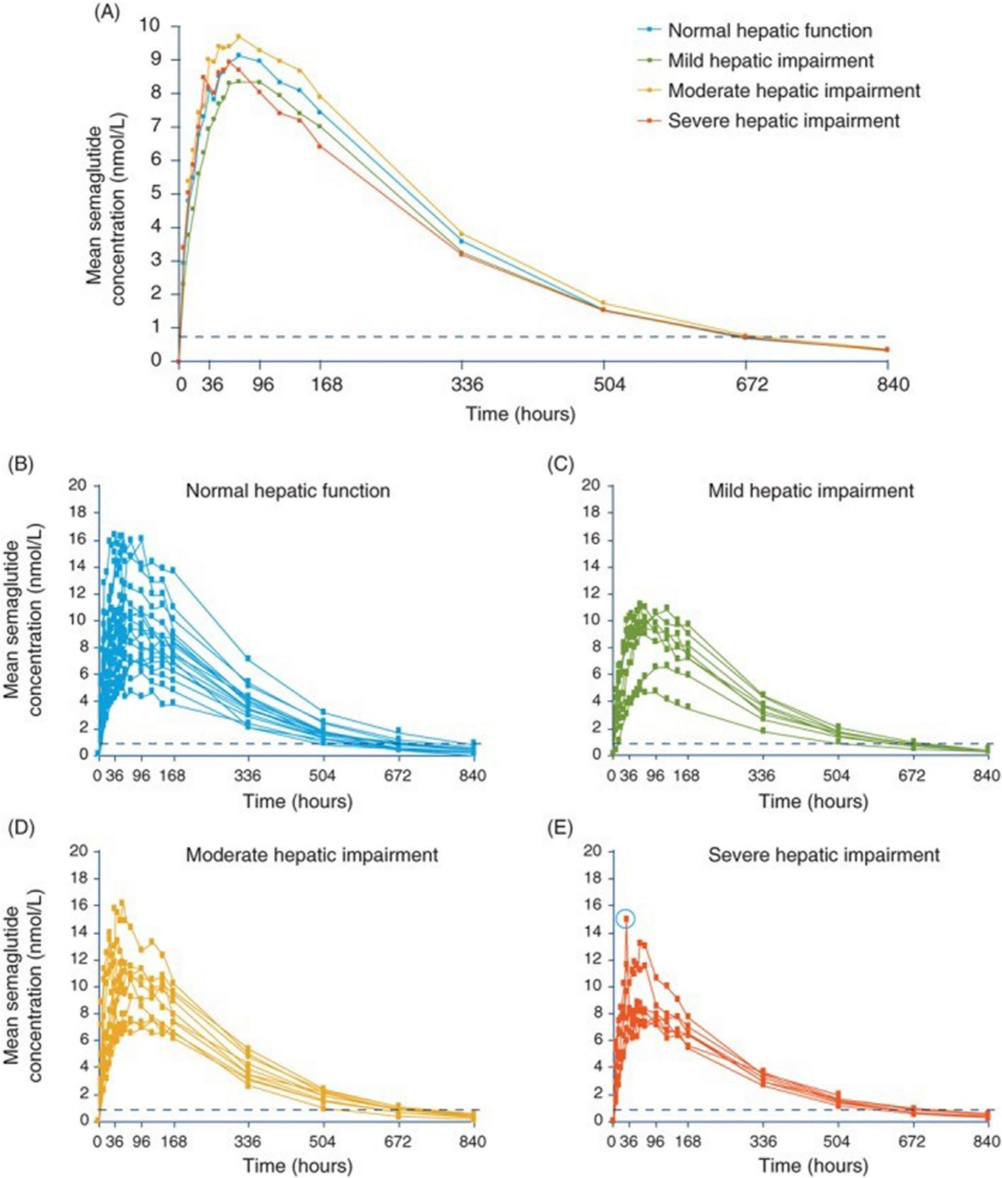
Plasma semaglutide concentration–time profiles in participants with normal hepatic function and those with hepatic impairment after a single dose of subcutaneous semaglutide 0.5 mg. A, Geometric mean profiles. B–E, Individual participant profiles. The circled data point in E represents the outlier excluded from the sensitivity analysis for Cmax. Data are geometric means. Values below the lower limit of quantification (represented by the dotted line) were imputed [85]

The LC-MS/MS procedure was also applied for the determination of semaglutide in human plasma in studies investigating the pharmacokinetics, safety, and tolerability of the drug after oral administration in subjects with and without renal impairment [77]. The same chromatographic condition was also used in a study evaluating the effects of oral semaglutide on the pharmacokinetics of lisinopril, warfarin, digoxin, and metformin in healthy subjects [78]. LC-MS/MS was also applied in another semaglutide pharmacokinetics study conducted on both healthy subjects and those with type 2 diabetes [79]. For assessing the safety and pharmacokinetics of oral semaglutide in healthy subjects and subjects with type 2 diabetes, the drug was measured in plasma after protein precipitation using LC-MS/MS with a lower Limit of quantification of 0.73 nmol/L [80]. Petri et al. applied the same chromatographic conditions for the analysis of semaglutide to identify clinically relevant covariates influencing semaglutide exposure [81]. The LC-MS/MS procedure was also applied in a study of the pharmacokinetics, pharmacodynamics, and safety of once-weekly subcutaneous semaglutide in healthy Japanese and Caucasian male subjects [82]. Jensen et al. applied the same LC-MS/MS conditions to analyze semaglutide in plasma levels in a study examining the drug’s pharmacokinetics and tolerability in patients with hepatic impairment [83]. Semaglutide concentration time-profiles mesured using the LC-MS/MS procedure are shown in Fig. 10.

To explain the influence of tablet erosion kinetics on the absorption of oral semaglutide, as well as the effect of water volume administered with dosing on tablet erosion kinetics and pharmacokinetic properties of oral semaglutide, the same LC-MS/MS procedure was applied [84]. Semaglutide was also determined in human plasma by the previously described LC-MS/MS procedure for studies evaluating the possible effect of inflamed stomach mucosa and/or an oesophageal disorder on the pharmacokinetics, safety and tolerability of oral semaglutide in subjects with type 2 diabetes and chronic gastritis and/or gastroesophageal reflux disease [85].

The concentration of semaglutide in plasma samples after protein precipitation was determined on an ACE C4 column with mobile phase containing acetonitrile, water, formic acid and ammonium acetate [86].

In another study, semaglutide was analyzed in breast milk from healthy mothers after subcutaneous administration of the drug [87]. Semaglutide was quantified using LC-MS on a Magic3 C-18 column. The mobile phase contained acetonitrile, water, and 0.1% formic acid. Detection was performed on a quadrupole mass spectrometer operated in positive ion mode. The LLOQ obtained by the procedure was 5.7 ng/mL.

To investigate the impact of production methods on the stability and impurities of semaglutide drug substances and products – and their potential impact on drug quality, efficacy, and safety – RP-HPLC analysis was conducted using a C18 column with a mobile phase consisting of acetonitrile, isopropyl alcohol, water and 0.08 M ammonium phosphate [88]. In the procedure, semaglutide was detected at a wavelength of 280 nm. LC-MS and LC-MS/MS analyses were performed using a Waters Acquity^®^ CSH C18 column with a mixture of water, acetonitrile, and 0.1% formic acid.

Semaglutide is occasionally determined by HPLC coupled with spectrophotometric detection. A high-performance liquid chromatography (HPLC) was used to quantify semaglutide in lipid nanocapsules [89]. Chromatographic analysis was performed on a Kinetex EVO C18 column with a mobile phase containing acetonitrile, water, and trifluoroacetic acid. Semaglutide was detected at a wavelength of 220 nm. In the procedure, the Limits of detection and quantification were 1.8 ± 0.8 µg/mL and 5.3 ± 2.3 µg/mL, respectively.

Long-acting injectable semaglutide microcapsules were quantified by HPLC using a system containing a C8 column and a mixture of acetonitrile, water, and 0.1% trifluoroacetic acid [69].

Size-exclusion chromatography coupled with multi-angle laser light scattering was applied to analyze the aggregation properties of semaglutide, among others [90]. The analysis was performed on a Sepax Zenix-C column with an aqueous mobile phase containing 200 mM NaCl and 10 mM Tris–HCl at a pH of 7.4. Detection was performed using a UV detector operated at 280 nm. Lipophilicity was determined using a C18 column with a mobile phase containing acetonitrile and an aqueous solution of formic acid (pH = 2.6) or ammonium acetate buffers adjusted to pH 7.4.

Liquid chromatography combined with spectrophotometric detection and mass spectrometry was also rarely used. The analysis of semaglutide after synthesis was analyzed using ultra-high performance liquid chromatography-UV-mass spectrometry (UPLC-UV-MS) on a BEH130 C4 column with a mobile phase containing acetonitrile, water, and 0.1% trifluoroacetic acid [91]. UV detection was performed at 214 nm, while MS analysis was performed with electrospray ionization in positive ion detection mode.

## Advantages, disadvantages of using semaglutide and challenges and future research priorities

Semaglutide effectively reduces glycated hemoglobin (HbA1c) levels and body weight across a broad spectrum of patients with type 2 diabetes, while also demonstrating cardiovascular safety [92]. Semaglutide may also protect the kidneys from damage caused by high blood sugar levels, which can lead to chronic kidney disease and kidney failure.

The introduction of oral semaglutide marks a milestone in the treatment of type 2 Diabetes, offering patients a more comfortable treatment option. However, the oral bioavailability of semaglutide is only from 0.4 to 1% [93]. Semaglutide must be taken on an empty stomach in the morning. Any food, drink, or other drugs within six hours before or half an hour after administration of semaglutide must not be taken. A disadvantage of oral semaglutide is the frequent occurrence of gastrointestinal discomfort. The advantage of semaglutide administered by injection is lack of digestive system disorders and the possibility of administering the drug once a week.

Obesity is an independent risk factor for cardiovascular disease and a modulator of other risk factors, hence, weight-reducing therapies are important in managing of these risk.

Semaglutide treatment was associated with a reduction in relative and absolute body weight and had a beneficial effect on cardiovascular risk factors such as BMI, waist circumference and blood pressure [94].

Seddio et al. reported that semaglutide utilized associated with reduced postoperative complications following single-level posterior lumbar fusion for patients with type II diabetes [95].

Golub et al. reported that semaglutide has a beneficial effect hepatic steatosis, demonstrated by a reduction in fatty liver content [96].

In obese mice, semaglutide treatment improved lipid metabolism, reduced oxidative stress and inflammation, decreased lipid peroxidation, and protected cardiac functions [97].

The use of semaglutide, in addition to its benefits, especially in the treatment of diabetes, may cause some undesirable effects, especially if used improperly and in excessive doses.

Semaglutide is not recommended for people with type 1 Diabetes and Diabetic ketoacidosis. It also is not advised for people who are pregnant or breastfeeding, or for children under 18 years of age.

Due to the delay of stomach emptying caused by semaglutide, the absorption of other oral medications administered at the same time may be affected.

Role of semaglutide in exacerbate symptoms of gastroesophageal reflux disease in patients with existing diagnoses and may induce gastroesophageal reflux in patients who are genetically predisposed to it due to the delayed gastric emptying effects of GLP-1 agonists was also reported [98].

Contraindications to the use of semaglutide in patients with history of medullary thyroid cancer and multiple endocrine neoplasia syndrome type 2 was described [99].

Semaglutide treatment is associated with increases in heart rate of 1 to 4 beats per minute. For this reason,, pulse rate should be monitored routinely in patients taking semaglutide, and the medication should be stopped in those with sustained increases [99].

Pancreatitis was also reported as a rare complication of treatment by semaglutide [99].

There is currently a significant increase in the use of semaglutide, particularly for weight loss, which has unfortunately led to an increase in reports of medication errors and adverse events associated with the use of this drug. There are numerous reported cases of exceeding recommended dosages of the drug [100]. Patients who overdosed semaglutide experienced side effects lasting several days, including nausea, vomiting and abdominal pain.

Acosta et al. reported a case of an intentional overdose of semaglutide leading to pancreatitis and ultimately euglycemic non-diabetic ketoacidosis [101]. This is due to overstimulation by GLP-1 agonists, which can then lead to hyperplasia and subsequent pancreatitis. Acute pancreatitis can induce a systemic inflammatory response and may be responsible for beta cells dysfunction and associated insulin deficiency, resulting in diabetic ketoacidosis.

Semaglutide is associated with a risk of hypoglycaemia, however, due to the glucose-dependent mode-of-action of semaglutide, the risk is relatively small compared to other diabetes treatments [102]. Hypoglycemia was seen most often in patients taking concomitant other drugs especially sulfonylureas [103]. Chen et al. concluded on the basis of a meta-analysis that the combination treatment of semaglutide and basal insulin demonstrates significant improvements in glycemic control and reduction in body weight, without an increased risk of hypoglycaemia [104].

Rarely other unexpected effects of semaglutide are reported, including acute cholecystitis, paralysis of the abducens nerve, and positional vertigo [105].

Body weight is related to insulin resistance and glycaemic control in type 2 Diabetes mellitus. Patients with type 2 diabetes who are overweight or obese face increased risk of complications, including circulatory system diseases, liver diseases, and kidney diseases [106]. Semaglutide a GLP-1 receptor agonist is effective in promoting both weight loss and lowering blood glucose in the treatment of people with type 2 Diabetes. However, individuals with type 2 diabetes tend to lose less weight than those without diabetes. The reasons for this difference are not fully understood, but it is generally believed that the blood glucose-lowering effect of semaglutide, leading to reduced urinary glucose loss, may result in relative energy retention, which may reduce weight loss [107]. Additionally, the concomitant use of semaglutide with other type 2 diabetes medications, such as insulin or sulfonylureas, which promote weight gain may also reduce the weight loss effect. Reduced weight loss in people with diabetes may also be explained by pancreatic β-cell dysfunction, as well as alterations in metabolic rate and energy expenditure often seen in type 2 Diabetes. However, there is still a need to explain why weight loss achieved with semaglutide treatment in people with type 2 Diabetes and obesity or overweight is usually lower than in people without type 2 diabetes. The exact role of semaglutide in the treatment of obesity should be determined in future studies including those with a large number of participants and a long follow-up period.

It may also be interesting to use combination therapy, taking advantage of the complementary pharmacological actions of semaglutide and other molecular bases. An example of a hybrid molecule is tirzepatide, which combines the effects of GLP-1 and GIP receptor agonism to maximize the efficacy of two incretins. A promising solution may also be the combination of GLP-1 receptor agonism with amylin receptor agonism (cagrilintide).

Currently, an obesity epidemic is observed in many countries and further research on the use of semaglutide in the treatment of obesity is indicated.

The exact nature of the interaction between semaglutide and thyroid function is still not fully understood, and further studies are needed to elucidate these mechanisms.

## Conclusions

Glucagon-like peptide-1 receptor agonists are an important class of glucose-lowering drugs. Semaglutide has emerged as a treatment option for type 2 diabetes, offering substantial improvements in health outcomes. As a GLP-1RA, semaglutide increases the secretion of insulin from pancreatic β-cells and supresses glucagon release from pancreatic α-cells. It is available in two forms: oral and injectable.Semaglutide has also exhibited the highest percentage of weight loss among all current anti-obesity medications. Moreover, the effects of semaglutide on blood glucose and body weight are associated with a reduction in cardiovascular risk.

Although gastrointestinal complaints are frequently reported (such as nausea, diarrhea, and vomiting), semaglutide continues to be an effective treatment option for Diabetes and obesity. Semaglutide is increasingly used for the treatment of type 2 diabetes, though its misuse for weight loss is also increasing.

There remains a need to develop safer and more convenient forms of administration of this drug.

The number of analytical procedures described in the literature for the determination of semaglutide by liquid chromatography remains limited. Semaglutide is most often determined using liquid chromatography coupled with tandem mass spectrometry. Chromatographic separation was often performed on C18 columns with a mobile phase containing acetonitrile, water, and formic acid. The drug was most often quantified in plasma samples.

## Data Availability

No datasets were generated or analysed during the current study.
